# Concurrent RAS and RAS/BRAF V600E Variants in Colorectal Cancer: More Frequent Than Expected? A Case Report

**DOI:** 10.3389/fonc.2022.863639

**Published:** 2022-04-07

**Authors:** Veronica Zelli, Alessandro Parisi, Leonardo Patruno, Katia Cannita, Corrado Ficorella, Carla Luzi, Chiara Compagnoni, Francesca Zazzeroni, Edoardo Alesse, Alessandra Tessitore

**Affiliations:** ^1^ Department of Biotechnological and Applied Clinical Sciences, University of L’Aquila, L’Aquila, Italy; ^2^ Center for Molecular Diagnostics and Advanced Therapies, University of L’Aquila, L’Aquila, Italy; ^3^ Department of Life, Health and Environmental Sciences, University of L’Aquila, L’Aquila, Italy; ^4^ Medical Oncology Unit, St. Salvatore Hospital, L’Aquila, Italy; ^5^ Medical Oncology Unit, “Giuseppe Mazzini” Hospital, Teramo, Italy

**Keywords:** colorectal cancer, concurrent *RAS/BRAF* variants, case report, mutation frequency, clinical–pathological features

## Abstract

The assessment of *RAS* and *BRAF* mutational status is one of the main steps in the diagnostic and therapeutic algorithm of metastatic colorectal cancer (mCRC). Multiple mutations in the BRAF and RAS pathway are described as a rare event, with concurrent variants in *KRAS* and *BRAF* genes observed in approximately 0.05% of mCRC cases. Here, we report data from a case series affected by high-risk stage III and stage IV CRC and tested for *RAS* and *BRAF* mutation, treated at our Medical Oncology Unit. The analysis of *KRAS*, *NRAS* (codons 12, 13, 59, 61, 117, 146), and *BRAF* (codon 600) hotspot variants was performed in 161 CRC tumors from August 2018 to September 2021 and revealed three (1.8%) patients showing mutations in both *KRAS* and *BRAF* (V600E), including two cases with earlier CRC and one with metastatic disease. We also identified one patient (0.6%) with a mutation in both *KRAS* and *NRAS* genes and another one (0.6%) with a double *KRAS* mutation. Notably, the latter was characterized by aggressive behavior and poor clinical outcome. The mutational status, pathological features, and clinical history of these five CRC cases are described. Overall, this study case series adds evidence to the limited available literature concerning both the epidemiological and clinical aspects of CRC cases characterized by the presence of concurrent *RAS/BRAF* variants. Future multicentric studies will be required to increase the sample size and provide additional value to results observed so far in order to improve clinical management of this subgroup of CRC patients.

## Introduction

Colorectal cancer (CRC) is the second most commonly diagnosed cancer in women and the third most commonly diagnosed cancer in men worldwide, accounting for about 10% of all tumors and 9% of cancer-related deaths in both sexes (1).

CRC incidence varies greatly in different geographical areas, with highest rates observed in Europe, Australia/New Zealand, Northern America, and Eastern Asia, and lowest rates in Africa and Southern Asia. While the variability in incidence rates can be mainly attributable to factors including dietary patterns, obesity, and lifestyle habits, survival rates are closely related to the improvement of screening and early detection programs and to the increase in therapeutic options, particularly in the metastatic setting (1).

Rat sarcoma (RAS) family proteins (KRAS, NRAS, and HRAS) and v-raf murine sarcoma viral oncogene homolog B1 (BRAF) are key regulators of the epithelial growth factor receptor (EGFR) signaling pathway (2), playing an essential role in biological processes including cell proliferation, survival, and differentiation and in cancer development, progression, and metastasis (3, 4).

Pathogenic variants in *RAS* genes, particularly *KRAS*, the most prominent member of RAS family proteins, are reported in about 40% of metastatic CRCs (mCRC) (5), 85% of which have *KRAS* missense gain of function mutations, mainly involving codons 12, 13, and 61 and, more rarely, codons 59, 117, and 146 (6). Similarly, *NRAS* mutations mainly occur in codons 12, 13, and 61 and are observed in approximately 2%–7% of mCRCs (7, 8). On the other hand, *BRAF* activating mutations occur in about 8%–12% of CRC cases, and approximately 90% of *BRAF* mutations involve codon 600 (9).

In addition to being considered a hallmark in CRC pathogenesis, the identification of these gene alterations has also relevant clinical implications, representing potentially actionable driver mutations and prognostic-predictive biomarkers (8). In particular, *KRAS* and *BRAF* V600E mutations confer poor prognosis and lack of response to anti-epidermal growth factor receptor (EGFR) monoclonal antibodies (i.e. cetuximab, panitumumab) in combination with chemotherapy in the metastatic disease (10, 11). Therefore, the assessment of *RAS* and *BRAF* mutational status is a main step in the diagnostic and therapeutic algorithm of CRC, both for predictive and prognostic aims, particularly in the metastatic disease setting, in order to support physicians in properly choosing the best treatment strategy as first and subsequent lines of treatment both in left- and right-sided tumors (12–16).

Although originally considered mutually exclusive, it is now demonstrated that the presence of multiple variants in the BRAF and RAS pathway can be possible, although a rare event, since concurrent *KRAS/BRAF* mutations are reported in approximately 0.05% of mCRC cases (17). Likewise, concomitant *KRAS/NRAS* mutations and double *KRAS* mutations have only rarely been described (18). However, consistent with recent findings (19), data from our unit show that this scenario could be more recurrent than expected.

To date, the biological and clinical significance of these peculiar genetic assets in CRC remains unclear.

Here, we describe the mutational status, pathological features, and clinical history of five CRC cases characterized by concurrent *BRAF* V600E*/KRAS* (three patients), *KRAS/NRAS* (one patient), and double *KRAS* (one patient) variants.

## Methods and Cases Description

One hundred sixty-one high-risk stage III and stage IV CRC cases referred to ASL1 Abruzzo Avezzano-L’Aquila-Sulmona were genotyped, according to the current Italian AIOM guidelines for CRC tumor (https://www.aiom.it/wp-content/uploads/2020/10/2020_LG_AIOM_Colon.pdf, https://testbiomolecolari.it/sites/default/files/private/attachments/20150508_Raccomandazioni_RAS.pdf), between August 2018 and September 2021 to investigate *KRAS*, *NRAS*, and *BRAF* hotspot mutations for potential anti-EGFR targeted therapy (i.e., cetuximab and panitumumab). All the five cases here described are from the Medical Oncology Unit of the S. Salvatore Hospital of L’Aquila. Genomic DNA was extracted from five 10-μm-thick microdissected formalin-fixed paraffin-embedded (FFPE) tumor sections [a double extraction was performed using ZYMO DNA FFPE (ZYMO research) and QIAamp DSP DNA FFPE (Qiagen) Tissue Kit] according to the manufacturer’s instructions. DNA was quantified with Qubit dsDNA HS Assay Kit (Invitrogen, Waltham, MA, USA), and the overall DNA yield was in a range of 30–50 ng/µl in a terminal volume of 25 µl.

Hotspot mutation analysis in the *KRAS*, *NRAS*, and *BRAF* genes was carried out using the CE-IVD qRT-PCR EasyPGX^®^ ready BRAF, KRAS, and NRAS kits (Diatech Pharmacogenetics, Jesi, IT), based on the allele-specific PCR method, and run on the Easy PGX qPCR instrument (Diatech Pharmacogenetics). The Diatech tests allow to examine the most common mutations in codons 12, 13, 59, 61, 117, and 146 of *KRAS* (95% of *KRAS* mutations described in CRC, as reported in Cosmic Database) and *NRAS* (90% of *NRAS* mutations described in CRC, as reported in Cosmic Database) and in codon 600 of *BRAF* gene (95% of *BRAF* mutations described in CRC, as reported in Cosmic Database), with a limit of detection up to 0.5%, without making it possible to exactly determine the corresponding allele frequency.

The used kits consist of several assays, each of which contains primers and probes for the detection of a specific mutated sequence (target probe, labeled with FAM) and endogenous control gene (control probe, labeled with HEX) to verify the accuracy of the amplification procedure.

Mutation analysis was performed in duplicate by using tumor DNA samples obtained from different extractions.

When possible, detected mutations were further validated by using the quantitative reverse transcription PCR (qRT-PCR) EntroGen KRAS/BRAF and NRAS mutation analysis kits (EntroGen) and/or by automated Sanger sequencing (**Supplementary Table S1**). All biomolecular technical data were recovered from the genotyping reports, where it was also stated that all the laboratory procedures were performed, according to the current guidelines, in dedicated rooms and instruments to avoid contaminations.

Clinico–pathological and molecular data of the five cases described in this study are summarized in **Table 1**.

**Table 1:** Clinico-pathological and molecular data of the five CRC cases with concurrent *RAS* and *BRAF/RAS* mutations.

Case ID	Sex	Age	Primary tumor site	Stage	Metastases	MMR/MSI status	RAS mutation (codon)	BRAF mutation (codon)	Adjuvant therapy/first-line treatment	Best response	OS (months)
1	Male	71	─	III	─	uncertain	KRAS G12D	BRAF V600E	Adjuvant XELOX	NA	32 (alive)
2	Male	66	right	III	─	pMMR	KRAS G12V	BRAF V600E	Adjuvant XELOX	NA	20 (alive)
3	Female	32	left	III	Yes	pMMR	KRAS G12C	BRAF V600E	FOLFIRI, Bevacizumab	na	Na
4	Female	43	right	IV	Yes (bone, muscle tissues)	pMMR	KRAS G12C; NRAS A146T/V	Wild-type	Oxaliplatin, Bevacizumab, Irinotecan, 5FU	PD	24 (alive)
5	Female	82	right	IV	Yes (liver)	pMMR	KRAS G12A; KRAS A146V	Wild-type	Oxaliplatin, Bevacizumab, Capecitabine	PD	8

MMR/MSI, mismatch repair/microsatellite instability; pMMR, proficient MMR; XELOX, chemotherapy regimen consisting of capecitabine plus oxaliplatin; 5FU, 5-Fluorouracil; FOLFIRI, chemotherapy regimen consisting of irinotecan, 5-Fluorouracil and leucovorin; PD, progressive disease; OS, overall survival; NA, not applicable; na, not available.

### Cases 1–3: Concurrent *KRAS/BRAF* Variants

A 71-year-old patient (case 1) without significant comorbidities or familiarity for neoplasms came to our first observation after surgery for stage III rectal cancer [pT3 pN1a according to tumor–nodes–metastasis (TNM) classification] in May 2019. Immunohistochemistry testing for mismatch repair (MMR) status showed uncertain results. *RAS/BRAF* mutational analysis from tumor surgical specimen revealed the presence of KRAS G12D and BRAF V600E variants. After post-surgical staging with computed tomography (CT) with no evidence of distant metastases, the patient underwent pharmacogenomic testing for dihydropyrimidine dehydrogenase (DPD), which evidenced no polymorphism. Patient was therefore scheduled for adjuvant systemic treatment with XELOX regimen for three cycles from May to July 2019, followed by chemo-radiotherapy treatment with concomitant Capecitabine from July to September 2019. He received a subsequent chemotherapy treatment with XELOX regimen for further three cycles ending in November 2019. The patient had no relevant treatment-related adverse events, with the exception for G1 neurotoxicity and diarrhea. At 32 months of follow-up, the patient evidenced no disease relapse.

**Figure 1:**
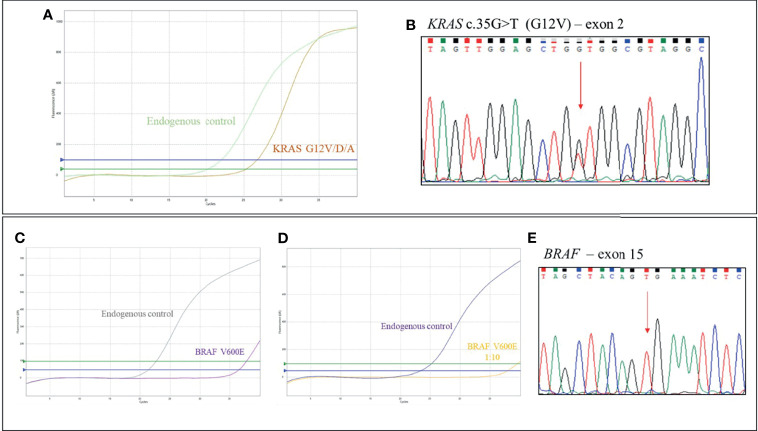
Case 2, *KRAS/BRAF* genotyping: **(A)** qRT-PCR results using EasyPGX^®^ KRAS kit showing the KRAS G12V/D/A (c.35G>H) variant in tumor DNA. The assay detects but not distinguishes the c. 35G>T (G12V), c. 35G>A (G12D) and c. 35G>C (G12A) mutations. Each assay contains primers and fluorescent probes allowing the simultaneous detection of target (FAM) and endogenous control gene (HEX). Threshold fluorescence for FAM (blue line) and HEX (green line) are shown. The assay clearly shows the presence of the variant, based on fluorescence signals quality and cutoff parameters. **(B)** Electropherogram from direct sequencing confirming the presence of *KRAS* c.35G>T (G12V) mutation in tumor DNA. **(C)** qRT-PCR results using EasyPGX^®^ BRAF kit showing the BRAF c. 1799T>A or c. 1799_1800TG>AA (indistinguishable) (V600E) mutation in tumor DNA. The mutation was undetectable by Sanger sequencing, **(E)** but it was still detected by qRT-PCR, respecting all quality and cutoff parameters, when tumor DNA was used at 1:10 dilution **(D)**.

A 66-year-old man (case 2), with positive history for ischemic heart disease and chronic obstructive pulmonary disease, was diagnosed with stage III right-sided CRC in April 2020. Immunohistochemistry testing for MMR showed a pMMR status (proficient MMR). *RAS/BRAF* mutational analysis from the tumor surgical specimen revealed the presence of KRAS G12V and BRAF V600E variants (**Figure 1**). Notably, most probably due to the low allele frequency of the BRAF V600E mutation, this alteration was undetectable by Sanger sequencing (**Figure 1E**), but it was still detected by qRT-PCR, with respect to all quality and cut-off parameters, in a 1:10 tumor DNA dilution (**Figure 1D**). After post-surgical staging with CT scan with no evidence of residual disease or distant metastases, the patient received adjuvant chemotherapy with XELOX regimen for 6 months, with dose reduction due to peripheral neuropathy. The patient is currently under follow-up, without any sign of disease relapse (DFS, 20 months).

A 32-year-old woman, with no relevant comorbidities, was diagnosed with stage III (pT3 pN2b according to TNM classification) left-sided pMMR CRC. Genetic analysis revealed the coexistence of KRAS G12C and BRAF V600E variants. The patient was scheduled for systemic adjuvant treatment, which the patient decided to start at another institution. Twenty months after curative surgery, disease relapsed, and the patient started first-line systemic treatment with FOLFIRI regimen combined with bevacizumab. Additional follow-up data are not available as the patient moved to another hospital.

### Case 4: Concurrent *KRAS/NRAS* Variants

A 43-year-old woman arrived at our unit with diagnosis of bone and muscle tissue metastases by an adenocarcinoma likely to be related to a previous sigmoid stage I (pT2 pN0 according to TNM classification) CRC after 4 years from curative surgery. The patient reported previous diagnosis of peritoneal lymphangiomatosis and bacterial sacroileitis. Immunohistochemistry testing for MMR showed a pMMR status. *RAS/BRAF* mutational analysis revealed the presence of KRAS G12C and NRAS A146T/V, the latter indistinguishable. DNA sample was not enough to perform Sanger sequencing analysis to ascertain the nature of *NRAS* variant. Preliminary pharmacogenomic DPD analysis showed no relevant polymorphism. The patient received treatment with FIr-B/FOx regimen as first-line treatment (20) from January 2020 for 6 months.

According to RECIST criteria, the patient showed partial response and stable disease at the CT scan performed after 3 and 6 months, respectively. The tolerance to the treatment was well, with the exception of G1 thrombocytopenia and palmar–plantar erythrodysesthesia, thus treatment with oxaliplatin was discontinued.

After 6 months of treatment, the patient underwent maintenance therapy with bevacizumab and fluoropyrimidines. At the last radiological re-evaluation in September 2021, a locoregional and lung disease progression occurred, after 14 months of maintenance treatment. In addition, the patient reported the occurrence of bone pain at the sacral spine level together with increase in the tumor markers carcinoembryonic antigen (CEA) and carbohydrate antigen (CA19-9). From October 2021, the patient was subjected to second-line treatment with FOLFIRI plus bevacizumab, still ongoing, with evidence of stable disease at the CT evaluation performed on January 2022.

### Case 5: Double *KRAS* Variants

**Figure 2:**
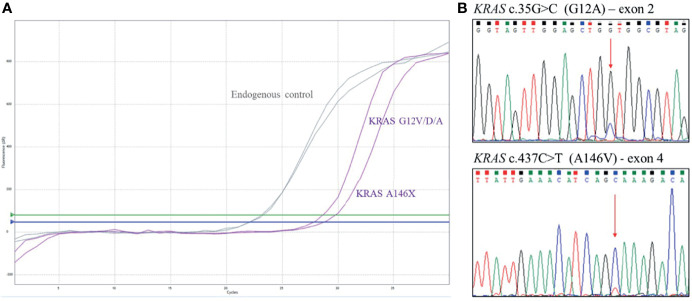
Case 5, *KRAS* genotyping. **(A)** qRT-PCR results using EasyPGX^®^ KRAS kit showing the double *KRAS* variant in tumor DNA. The assay KRAS G12V/D/A detects but not distinguishes the c. 35G>T (G12V ), c. 35G>A (G12D) and c. 35G>C (G12A) mutations. The assay KRAS A146X detects but not distinguishes the c. 436G>A (A146T), c. 436G>C (A146P) and c. 437C>T (A146V) variants. Each assay contains primers and fluorescent probes allowing the simultaneous detection of target (FAM) and endogenous control gene (HEX). Threshold fluorescence for FAM (blue line) and HEX (green line) are shown. The assays clearly show the presence of the variants, based on fluorescence signals quality and cutoff parameters. **(B)** Electropherogram from direct sequencing confirming *KRAS* variants c.35G>C (G12A) and c.437C>T (A146V).

An 82-year-old woman, with history of hypertension, was diagnosed with stage IV, right-sided, mCRC with liver metastases; CEA biomarker was already positive at diagnosis. Immuno-molecular investigations highlighted pMMR and the presence of two *KRAS* variants on exon 2 (G12A) and exon 4 (A146V), as confirmed by Sanger sequencing analysis (**Figure 2**).

**Figure 3:**
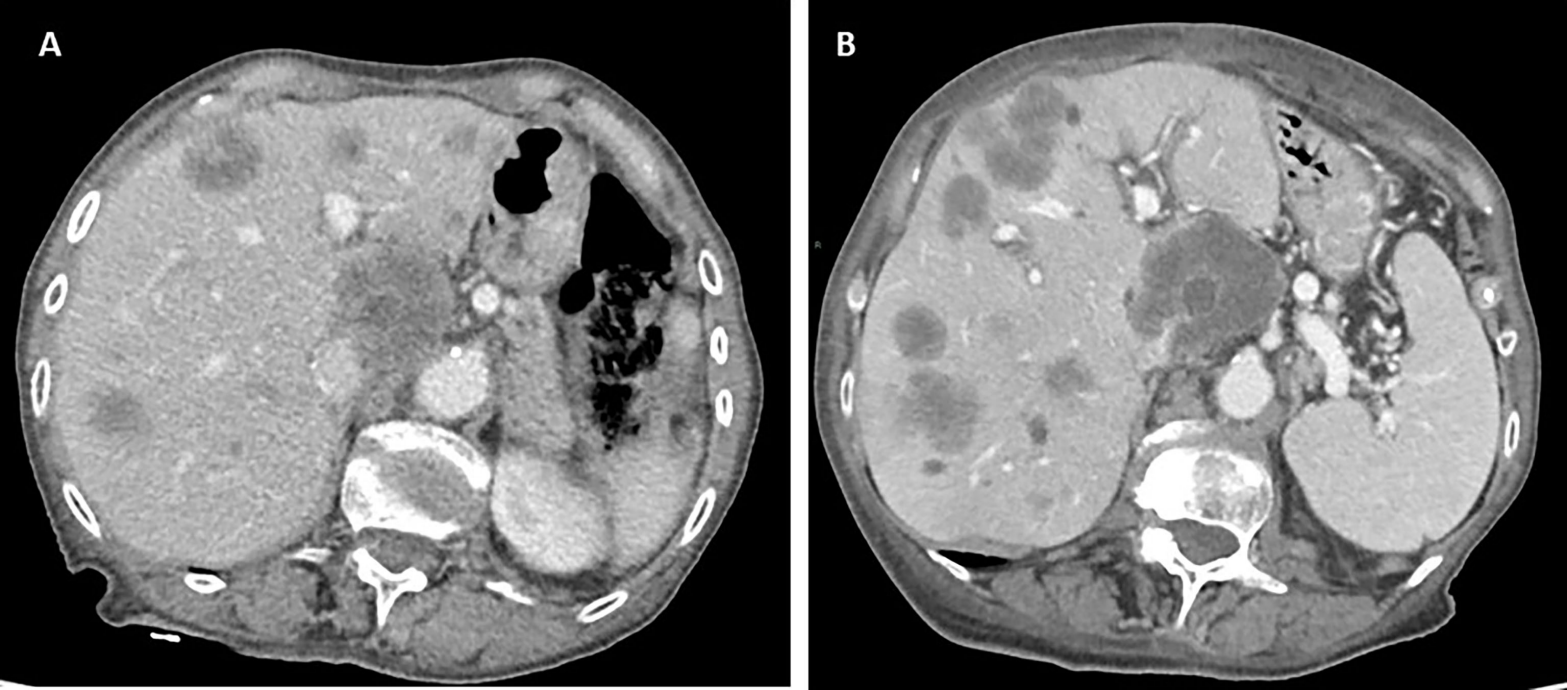
Computed tomography (CT) scan evaluation in patient 5 before **(A)** and after **(B)** 3 months of systemic treatment, showing rapid disease progression to the liver.

After disease staging with CT scan, the patient was scheduled for systemic treatment with biweekly Xelox plus bevacizumab with reduced dose according to age and performance status. Preliminary pharmacogenomic DPD analysis showed no significant polymorphisms. During treatment, the patient reported G1 gastrointestinal toxicity, hypertension, and asthenia. Instrumental re-evaluation with CT after 3 months of systemic treatment showed liver progression (according to RECIST criteria) (**Figure 3**). Upon evaluation of general conditions, the patient discontinued the systemic treatment and continued with exclusive supportive care. Overall, patient showed extremely rapid disease progression in 6 months and died 8 months after diagnosis.

## Discussion

In the context of precision medicine, the assessment of *RAS* and *BRAF* mutational status is essential for the use of monoclonal anti-EGFR therapies, due to their predictive role for resistance to this therapeutic approach (10, 11). In addition, the increase in molecular data and the deepening of their oncogenic function is providing further information of biological and clinical utility that may also lead to the development of new targeted drugs.

Concurrent variants affecting the same molecular pathway are generally mutually exclusive, indicating that the presence of multiple *RAS/BRAF* mutations in CRC patients can be considered a rare event (18).

In our cohort of CRC patients, *RAS/BRAF* mutational analysis revealed concurrent *KRAS/BRAF* variants in three (1.8%) cases (all BRAF V600E), *KRAS/NRAS* variants in one (0.6%) patient, and double *KRAS* variants in another one (0.6%).

To date, only about 30 mCRC cases with concomitant *BRAF/KRAS* mutations have been described in the literature (17), with a reported incidence, in two large series of mCRCs, of 0.064% and 0.2% (21, 22). Recently, concurrent *BRAF* and low-allele frequency *RAS* mutations were detected in four out of 581 mCRC cases, with a frequency of 0.7% (23). Of note, the reported frequencies generally refer to BRAF codon 600, mainly V600E variant, which accounts for about 90% of all *BRAF* mutations detected in CRC (9). Based on the molecular mechanism, kinase activity, and sensitivity to inhibitors, three different categories of *BRAF* mutations have been described: (i) RAS-independent kinase-activating V600 mutations that act as monomers (class I), (ii) RAS-independent kinase activating non-V600 mutations that signal as dimers (class II), and (iii) RAS-dependent kinase impaired non-V600 mutations that function as heterodimers. Unlike the other categories, class III *BRAF* variants are characterized by the amplification of ERK signaling through the activation of upstream tyrosine kinase receptors or by the presence of RAS mutations, thus showing a more likely co-occurrence with RAS alterations (24, 25).

Only few studies described the presence of concurrent *KRAS/NRAS* mutations in CRC, with estimated frequencies ranging from 0.1% to 0.9% (7, 18, 26–29); in particular, concurrent *KRAS/NRAS* mutations were detected in 11 of 1,294 (0.9%) European CRC patients (26), 8 of 1,110 (0.7%) Chinese cases (28), and 3 of 2,764 (0.1%) American CRC patients (7).

In a review by Macedo et al. (30), 71 cases showing double KRAS mutation were reported, with an estimated 1% average incidence (range, 0.4%–8.3%).

More recently, studies on larger CRC cohorts displayed *KRAS* double mutation frequency of 0.7% (8/1,110) in Chinese CRC patients (28) and 0.3% (2/744) in American population (18). Most of the reported double KRAS mutations occurred in a single codon, mainly codon 12 or in the two codons 12 and 13; to our knowledge, the combination of double mutation in *KRAS* codons 12 and 146, as for case 5, has only been described in another CRC patient (31).

Overall, the methods used to perform mutational analysis mainly included PCR-based techniques, Sanger sequencing, and next-generation sequencing (NGS). However, in several studies, in-depth data regarding the technique used are missing.

Based on the above-mentioned literature, concurrent *RAS/BRAF* variants have been described with low frequency; furthermore, the estimated incidence is extremely variable, and the fraction of early CRCs and mCRCs is not always clear among the analyzed specimens.

Interestingly, in a recent work using NGS, concurrent *KRAS/BRAF*, *KRAS/NRAS*, and double *KRAS* variants were identified in 1.8% (4/219), 0.9% (2/219), and 2.7% (6/219) cases, respectively, with one additional patient (1/219, 0.4%) showing three different *KRAS* mutations (19). Authors attributed these higher mutation frequencies to the higher sensitivity of NGS analysis with respect to standard methods (i.e., Sanger sequencing and conventional PCR), being able to detect genetic alterations even at very low allele frequency (up to 0.06%).

It is well known that the ability to specifically, sensitively, and accurately detect low-allele frequency somatic alterations in tumor DNA strongly depends on the method used for the analysis (32): in this context, NGS technologies represent the most powerful tool for this purpose, and their increasing use will allow to more carefully assess the incidence of the peculiar conditions here described, most probably higher than currently estimated (33, 34).

On the other hand, the introduction into clinical practice of increasingly sensitive mutational analysis technologies, such as NGS or qRT-PCR, has highlighted the matter of the minimum percentage of mutation associated with resistance to monoclonal anti-EGFR drugs. In the phase III CRYSTAL study, different RAS variant frequency thresholds were evaluated, and a possible benefit from anti-EGFR therapy was described in patients with mutant allele frequency included between 0.1% and 5% (35). Consistent with this finding, recent studies have confirmed that the threshold of 5% could better discriminate between anti-EGFR sensitive and resistant patients (23, 36).

The three CRCs harboring concomitant *KRAS/BRAF* V600E mutations were all diagnosed with stage III CRC, and one of them developed metastatic disease 20 months after diagnosis.

Therefore, we can speculate that the coexistence of *KRAS/BRAF* V600E mutations may not be strictly related to tumor progression and acquired resistance to anti-EGFR therapy (37), being detectable also in earlier stages of the disease. This consideration is consistent with the concept of intra-tumor heterogeneity, which characterizes solid neoplasms and the existence of subpopulations with peculiar somatic gene lesions within the same tumor (38).

To date, it is still unclear whether the presence of concurrent *RAS/BRAF* V600E variants could affect tumor behavior and progression and which of them could play, possibly, a predominant role (2).

However, *RAS/BRAF* mutant tumors seem to show different genetic signatures (39), suggesting that these multiple mutations could play a synergistic role in tumor development and progression by activating different signaling pathways (40, 41).

Moreover, concomitant *RAS/BRAF* (mainly V600E) variants are generally described to be associated with more advanced tumor stage, locoregional/distant metastases, and worse clinical outcome in CRC patients (40), although these findings have not been confirmed by further studies (22).

However, even if in a small number of cases, Afrăsânie et al. (17) reported that the median overall survival of mCRC patients with both *KRAS* and *BRAF* mutations (almost all affecting codon 600) was about 30% lower than the survival observed in the general mCRC population.

In consideration of these premises, due to the limited number of CRCs with concurrent *KRAS/BRAF* variants described in the literature, no conclusive pathological and clinical considerations can be deduced (17, 22).

So far, two of the cases here described have not shown tumor progression or resistance to treatment (32 and 19 months follow-up for cases 1 and 2, respectively); however, we cannot exclude that the presence of concurrent *KRAS/BRAF* V600E variants may result in a more aggressive tumor behavior and possible future therapy resistance.

Preclinical data highlighted different oncogenic effects of mutant *KRAS* and *NRAS* [principally the c.35G>A (G12D) variant], the combination of which may promote tumor development and/or progression. KRAS aberrant activity was found mainly associated with the regulation of cell proliferation, while NRAS was predominantly involved in cell survival (42, 43).

Although the possible functional consequences of concomitant *KRAS/NRAS* variants other than G12D, including the combination identified in case 4—*KRAS* c.34G>T (G12C) and *NRAS* c.436G>A/c.437G>T (A146T/V)—are still unknown, it was suggested that this specific genetic asset might provide a selective advantage in cancer cells over the presence of only one of the two mutations (29). This case was characterized by bone and muscle metastases, and after a partial response to first-line chemotherapy plus bevacizumab, the patient showed progression of local and pulmonary disease.

In CRC, an association between the presence of multiple *KRAS* variants and advanced clinical stage was reported (44). Several CRC cases with double *KRAS* lesions described in the literature were also characterized by an aggressive disease (30).

Consistent with these findings, our 82-year-old case 5, showing two *KRAS* variants in codons 12 and 146, was diagnosed with stage IV mCRC (liver metastases).

According to the current clinical guidelines for the fist-line treatment of metastatic CRC, the patient was treated with a combination of chemotherapy and Bevacizumab (45). Notably, case 5 was characterized by poor clinical outcome (disease progression and death 8 months after diagnosis).

In a recent study, De Falco et al. (46) described two cases of CRCs at different stages (pT2N0M0 and pT4cN1cM1) characterized by the presence of two *KRAS* variants. Patients were treated as single *KRAS* mutant, and both progressed towards a metastatic disease. The authors suggested that double *KRAS* variants could have a potentially severe impact on the clinical outcome, highlighting the need to consider this specific genetic asset as a peculiar molecular feature, which could benefit from a closer follow-up, regardless of the stage, and could point towards a different, personalized therapeutic approach. Taken together, these results suggest that multiple *KRAS* mutations could represent a distinct and peculiar genetic asset, possibly associated with aggressive behavior and poor prognosis in CRC. Further investigations are needed to assess and confirm the possible prognostic role of these genetic alterations, Furthermore, from a therapeutic point of view, this could lead to the identification of a subset of high-risk patients who could possibly benefit from the adoption of different and more specific clinical strategies (46). Of note, *KRAS* represents not only the main predictive factor for monoclonal anti-EGFR therapy but also a druggable target gene, as demonstrated by the FDA approval of MRTX849 (Adagrasib), a novel inhibitor administered in presence of the KRAS G12C mutation (47, 48). In addition, the phase I clinical trial for another G12C inhibitor (AMG510-Sotorasib) is currently ongoing for non-small cell lung cancer (49, 50).

Overall, the identification of concurrent and multiple *RAS* and *RAS/BRAF* V600E variants in CRC highlights the intratumor heterogeneity and the presence of subclonal populations with different genetic features in some CRCs (38).

How and in what allele frequency range in these multiple alterations can influence the clinical course of the disease and whether their early detection can direct more targeted therapeutic approaches remain unclear and subject of additional studies (41, 51).

Due to the restricted size of specimens analyzed in our monocentric study, it is difficult to draw univocal conclusions to be transferred to clinical consideration. However, this study adds evidence to the limited available literature concerning both the epidemiological and clinical aspects of CRC cases characterized by concurrent or multiple *RAS/BRAF* variants.

## Conclusions

Future multicenter observational studies on larger cohorts of CRC patients are needed to better identify the real frequency of concurrent/multiple *RAS/BRAF* variants, shed light on their significance, and deduce information of clinical utility.

## Data Availability Statement

The original contributions presented in the study are included in the article/**Supplementary Material**. Further inquiries can be directed to the corresponding author.

## Ethics Statement

Ethical review and approval was not required for the study on human participants in accordance with the local legislation and institutional requirements. Written informed consent from patients for publication of this manuscript and any accompanying images was obtained by medical oncologists participating to the study. No data or images are specifically ascribable to any patient identity.

## Author Contributions

VZ, AP, LP, and CC: writing manuscript. AP, LP, KC, and CF: clinical–molecular data recovering, interpretation, association, curation, and description. VZ, CC, and CL: methodological description and figure composition. FZ: reading manuscript. EA and AT: conceptualization, writing, and editing manuscript. All authors contributed to the article and approved the submitted version.

## Funding

VZ is supported by PON-AIM 2014–2020 Research and Innovation funding and by grants from University of L’Aquila Research Project 2021 (“Avvio alla Ricerca”), Department of Biotechnological and Applied Clinical Sciences, University of L’Aquila Research Project 2021 and Carispaq Foundation L’Aquila 2020.

## Conflict of Interest

The authors declare that the research was conducted in the absence of any commercial or financial relationships that could be construed as a potential conflict of interest.

## Publisher’s Note

All claims expressed in this article are solely those of the authors and do not necessarily represent those of their affiliated organizations, or those of the publisher, the editors and the reviewers. Any product that may be evaluated in this article, or claim that may be made by its manufacturer, is not guaranteed or endorsed by the publisher.
