# Supplementary material for: Concurrent RAS and RAS/BRAF V600E Variants in Colorectal Cancer: More Frequent Than Expected? A Case Report
Source: Front Oncol. 2022 Apr 7;12:863639. doi: 10.3389/fonc.2022.863639 (PMC9022079; doi:10.3389/fonc.2022.863639)
Supplement: Supplementary file 1 [file Table_1.docx]

**Supplementary Table 1.** *KRAS, NRAS* and *BRAF* hotspot mutation analysis techniques and validation methods

| Case ID | Mutation | Main analisys | Validation/other analyses | | | |
| --- | --- | --- | --- | --- | --- | --- |
|  |  | qRT-PCR EasyPGX kits^a^ | qRT-PCR EasyPGX kits (DNA at the 1:10 dilution) | qRT-PCR EntroGen kits | qRT-PCR EntroGen kits  (DNA at the 1:10 dilution) | Sanger sequencing |
| #1 | KRAS G12D | ✓ |  | ✓ |  |  |
|  | BRAF V600E | ✓^b^ |  |  |  |  |
| #2 | KRAS G12V | ✓ |  |  |  | ✓ |
|  | BRAF V600E | ✓ | ✓ | ✓ | ✓ |  |
| #3 | KRAS G12C | ✓ |  | ✓ |  | ✓ |
|  | BRAF V600E | ✓ |  |  |  |  |
| #4 | KRAS G12C | ✓ |  | ✓ |  |  |
|  | NRAS A146T/V | ✓ |  | ✓ |  |  |
| #5 | KRAS G12A | ✓ |  |  |  | ✓ |
|  | KRAS A146V | ✓ |  |  |  | ✓ |

^a^performed in duplicate using tumor DNA samples obtained from different extractions

^b^performed in quadruplicate
